# Perchlorate: Health Effects and Technologies for Its Removal from Water Resources

**DOI:** 10.3390/ijerph6041418

**Published:** 2009-04-14

**Authors:** Asha Srinivasan, Thiruvenkatachari Viraraghavan

**Affiliations:** 1 Faculty of Engineering, University of Regina, Regina, SK, S4S 0A2, Canada; E-Mail: sriniash@uregina.ca; 2 Professor Emeritus, Faculty of Engineering, University of Regina, Regina, SK, S4S 0A2, Canada

**Keywords:** Perchlorate, Drinking Water, Toxicity, Health Effects, Treatment Technologies

## Abstract

Perchlorate has been found in drinking water and surface waters in the United States and Canada. It is primarily associated with release from defense and military operations. Natural sources include certain fertilizers and potash ores. Although it is a strong oxidant, perchlorate is very persistent in the environment. At high concentrations perchlorate can affect the thyroid gland by inhibiting the uptake of iodine. A maximum contaminant level has not been set, while a guidance value of 6 ppb has been suggested by Health Canada. Perchlorate is measured in environmental samples primarily by ion chromatography. It can be removed from water by anion exchange or membrane filtration. Biological and chemical processes are also effective in removing this species from water.

## 1. Introduction

The perchlorate anion consists of a tetrahedral array of oxygen atoms around a central chlorine atom. Perchlorate is usually found as the anion component of a salt most often associated with cations such as ammonium, sodium, or potassium. It is typically found in the form of perchloric acid and salts such as ammonium perchlorate, potassium perchlorate, and sodium perchlorate. Perchlorate is a strong oxidizing anion and has recently gained public attention following its discovery in well water and drinking water throughout the United States. Perchlorate is ubiquitous and has been found to be present in soil, vegetation, groundwater and surface water in a number of places. The non-volatile and stable nature of the perchlorate anion has allowed it to be present in drinking water aquifers. It is used as an oxidizer in flares, pyrotechnics, explosives, and numerous other applications, and the recent detection of environmental contamination has primarily been associated with its use in rocket propellants and missile motors. Despite the detection of perchlorate in groundwater in the 1950s, its toxicological properties have only recently been examined in detail [1,2]. The primary toxicity issue is associated with disruption of iodide uptake in the thyroid gland due to its similarity in ionic radius to iodide. Widespread presence of perchlorate in drinking water aquifers and its toxicological properties make perchlorate an emerging chemical of concern.

### 1.1. Properties of Perchlorate

Commonly used perchlorate compounds are all mostly solid salts at ambient temperature and consist of white or clear crystals (Table 1). Perchlorate salts have low vapour pressure and hence cannot volatilize under ambient conditions. Perchlorate salts are highly soluble in water and dissolved perchlorate anions do not tend to partition from aqueous to gas phase. This soluble, noncomplexing nature makes perchlorate highly mobile in the aqueous environment and does not allow sorption to soils or bioaccumulation. This also implies that perchlorate cannot be treated by precipitation or sorption, while its nonvolatile nature implies that it cannot be treated by air stripping.

Thermodynamic measurements indicate perchlorate anion as a strong oxidant, however slow kinetics generally limits its reactivity. The lack of reactivity results from high strength of chlorine-oxygen bonds, and the requirement that reduction proceed by removal of an oxygen atom, rather than by direct interaction of a reducing agent with a chlorine atom [2]. This capacity as a strong oxidant and its limited reactivity nature allows perchlorate salts to persist in the environment and they are hard to treat by chemical reduction, while biologically mediated reactions can effectively degrade them.

## 2. Uses of Perchlorate

The most commonly manufactured perchlorate compounds are ammonium, sodium and potassium perchlorate and perchloric acid. Various perchlorate-containing materials are used for specialized applications in chemical industries. Because of its exceptional oxidizing capacity, perchlorate is widely used by the industry, in the manufacture of munitions, explosives and fireworks, a few specific medicinal applications and in small amounts in other high volume consumer products. Perchlorate has been used medically to control hyperthyroid conditions and Graves disease [4,5]. Martino *et al.* [4] studied the effect of potassium perchlorate in patients with hypothyroidism. Short term administration of potassium perchlorate to six patients led to prompt restoration of euthyroidism. Most of the perchlorate-contaminated sites were once associated with rocket manufacturing or munitions use. Use of fireworks, flares and blasting agents has also resulted in environmental contamination [6]. A study conducted on surface water samples following a firework display, found a maximum perchlorate concentration of 44.2 μg/L, while the concentrations decreased towards the background level within 20 to 80 days [7]. Perchlorate may also be formed as a breakdown product of sodium hypochlorite, widely used as a bleach and can be formed incidently in corrosion control applications.

## 3. Environmental Fate and Transport

Perchlorate is highly soluble in water and stable under normal atmospheric conditions. Once a perchlorate compound is released into the environment, most often into water bodies, it gets dissolved in water, and due to its limited ability to adsorb on to surfaces, and/or gets transported through bulk movement of water and mixing processes. When the flow is less or if water becomes stagnant, perchlorate tends to diffuse into clay layers in an aquifer. Perchlorate may be biologically degraded by indigenous microorganisms present in water under anaerobic conditions [8]. Terrestrial and aquatic plants can take up perchlorate and accumulate in various tissues or degrade it in their leaves and branches [9].

## 4. Perchlorate in the Environment

### 4.1. Presence of Naturally Occurring Perchlorate

Most naturally occurring sources of perchlorate appear to be geographically limited to arid environments. These deposits tend to be low in concentration, except for the relatively high natural concentrations found in Chilean caliches and some potash ores [10]. The major Chilean source lies in the Atacama Desert, where the fertilizer is extracted from deposits of nitrate ores or brines [11]. The deposits contain perchlorate ranging from 0.03 to 0.1% or 300 to 1,000 mg/kg in soil [10]. Potash ore is mined and milled in Saskatchewan, Canada (silvite mineral), United States (New Mexico, hankite mineral from California), and Playa crusts from Bolivia [12]. The potassium chloride in these deposits originated in briny sea beds, similar to those of Chilean deposits. The concentrations of perchlorate in these deposits range from 25 to 2,700 mg/kg. Infrequent precipitation in arid environments minimizes the opportunity for perchlorate in nitrate and potash deposits to dissolve and migrate to groundwater. Hence, perchlorate would remain in these deposits in larger quantities than the ones located in regions of higher precipitation.

### 4.2. Theory of Origin of Natural Perchlorate

In addition to occurring in these deposits, a current theory concerning the origin of naturally occurring perchlorate in the environment focusses on natural atmospheric processes. This theory suggests that chloride, possibly in the form of sodium chloride from sea or land based chloride compounds blown in from the atmosphere, reacts with atmospheric ozone to create perchlorate. This process occurs over much of the earth and is analogous to nitrate formation in the atmosphere [13]. Photolysis of ozone in the presence of sea salt is predicted to form chlorine (Cl_2_) and hypochlorous acid (HOCl) in the marine environment [14]. During such reactions, formation of chlorate (ClO_3_^−^) has been proposed in the stratosphere [15]. The reactions were reexamined on the surface of the sodium chloride particles [16] and studies show that oxygenated products do form even on dry salt surfaces [17]. Chlorate has also been observed to form during ozonation of chloride (Cl^−^) containing natural waters. Dissolution of molecular chlorine (Cl_2_) in water may result in formation of hypochlorite radicals (OCl^−^) [18]. Addition of oxygen to chlorate (ClO_3_^−^) or fractionation of hypochlorite may result in the formation of perchlorate radical (ClO_4_^−^) [19]. These reactions are thermodynamically favourable but reasons for not forming appreciable amounts of perchlorate may be kinetic [19]. Given the precursor ingredients and sufficiently energetic conditions, it should be possible to induce formation of perchlorate. A recent theory suggests that in addition, there is a possibility that lightning may play a role in the creation of some atmospherically produced perchlorate [19]. Following atmospheric creation, perchlorate returns to the earth’s surface dissolved in precipitation. In arid environments, where the rate of deposition exceeds the rate of dissolution by ongoing precipitation, perchlorate can be incorporated into certain geologic formations [11].

### 4.3. Presence of Perchlorate in Natural Waters

Table 2 presents information on perchlorate release into the environment. Perchlorate has been detected in selected surface waters in the Great Lakes basin in Canada [20]. Of the 55 sites in the Great Lakes basin sampled and analyzed for the presence of perchlorate, it was detected at concentrations near the detection limit in two samples from Hamilton Harbour and six creeks/rivers in the Maitland Valley and the Upper Thames River watersheds. The two detections in the Harbour were thought to be related to the fireworks display which had occurred prior to sampling. Presence of low but detectable concentrations of perchlorate, approximately 1 μg/L, has also been confirmed in an unconfined aquifer located in southwestern Ontario, Canada [21]. Most of the perchlorate contamination that has been found in the United States has been associated with military activities or defense contractors. Considerable attention has been focused on Lake Mead and the Colorado River because of the ammonium perchlorate production facilities formerly located in Henderson, Nevada. So far, dense perchlorate occurrence in United States has been found in California, Texas, Massachusetts, Maryland, Oregon and New Jersey. Drinking water in about 26 states in the United States is believed to be contaminated with perchlorate with less than 12 μg/L concentration [2]. A recent study to determine the overall distribution of perchlorate in California drinking water sources found that the mean perchlorate level was 3.6 μg/L and among sources with positive perchlorate results, the mean level was 12 μg/L [22].

The occurrence of perchlorate in Japan in the Tone River Basin has been recently detected [23]. Perchlorate was found at high concentrations in the upper Tone River and its tributary, Usui River, and the maximum concentrations were 340 and 2,300 μg/L, respectively. The possible sources of perchlorate in the two areas were attributed to industrial effluents. It was shown that tap waters in the Tone River Basin were also widely contaminated with perchlorate. Widespread contamination of perchlorate in sludge, rice, bottled water and milk collected from different areas in China has been confirmed [24]. The concentration of perchlorate in sewage sludge, rice, bottled drinking water and milk was in the range of 0.56–379.9 μg/kg, 0.16–4.88 μg/kg, 0.037–2.013 μg/L and 0.30–9.1 μg/L, respectively.

### 4.4. Perchlorate in Food Supplies

The primary route of human exposure to perchlorate is via ingestion of perchlorate-contaminated water and food. Ingested perchlorate is readily and completely absorbed from the gastrointestinal tract and excreted rapidly primarily via the urine. Based on available literature, ingestion of perchlorate contaminated drinking water is one of the major exposure routes of concern, although ingestion of contaminated food and human milk are other potential sources of exposure (Table 3). When compared to ingestion, skin absorption and inhalation of perchlorate can be considered negligible exposure pathways.

## 5. Analytical Methodologies

The primary method of analyzing drinking water and environmental samples for perchlorate has been ion chromatography. The selectivity combined with the low detection limit makes this the optimal technique. EPA's Office of Ground Water and Drinking Water released Method 314 for the analysis of drinking water [35]. Method 314 (Ion chromatography) can be applied for aqueous samples with low dissolved solids and for chloride, sulfate, and carbonate concentrations less than 100 mg/L each. An aqueous reporting limit of 4 μg/L is achievable. This method has been validated in drinking water only and no guidance is provided for use with soils and biota. A similar method 9058 (Ion chromatography) has been issued by the EPA's Office of Solid Waste and Emergency Response [36].This method can be applied to aqueous and soil samples. The reporting limit of 0.5–1 μg/L is achievable. A number of forensic approaches have been proposed regarding perchlorate source identification and age dating. The general categories available for this purpose include association of perchlorate with known sources. Perchlorate associated with safety flares may be distinguished from an association of the perchlorate ion with strontium nitrate (75% by weight), sulfur (*<* 10%), and oil (*<* 10%). Stable isotopic analysis is also done to identify the sources of perchlorate. Use of triple-oxygen isotope ratios, ^17^O/^16^O and ^18^O/^16^O ratios, and ^37^Cl/^35^Cl ratio are proposed as a means for distinguishing between anthropogenic and naturally occurring sources of perchlorate [37,38]. Use of scanning electron microscopy and/or x-ray diffraction may provide a means to confirm that soil enriched with perchlorate is associated with evaporate deposits and or a marine depositional origin.

## 6. Regulatory Limits

Regulators use the results of research into toxicological effects and evaluations of potential exposures in the context of other scientific and non-scientific issues to derive enforceable standards and guidance levels for perchlorate. In Canada, preliminary analysis of groundwater and surface water samples show very low levels of perchlorate and it has only been found in one Canadian public drinking water supply at a concentration below 1 ppb [39]. Scientific studies and guideline development related to perchlorate are on-going in Canada and United States. Health Canada recommends a drinking water guidance value of 6 ppb, based on a review of existing health risk assessments from other agencies. In 1998, the U.S. EPA added perchlorate on the drinking water contaminant candidate list (CCL) based on its presence in drinking water supplies in the southwestern United States. In 2005, the United States National Academy of Sciences produced a report on the health implications of perchlorate ingestion [40], which has been used by the U.S. EPA to establish an oral reference dose (RfD) of 0.0007 milligrams perchlorate per kilogram body weight per day and a drinking water equivalent level (DWEL) of 24.5 μg/L. This value was based on a study conducted by Greer *et al.* [41] in which healthy men and women were administered perchlorate at 0.007 to 0.5 mg/kg-day for a 14 day period. DWEL are not enforceable standards. The agency, on January 2009 has issued an Interim Drinking Water Health Advisory (Interim Health Advisory) for exposure to perchlorate of 15 μg/L in water. The Interim Health Advisory for perchlorate was developed using EPA’s RfD and representative body weight, as well as 90^th^ percentile drinking water and national food exposure data for pregnant women in order to protect the most sensitive population identified by the National Research Council (NRC) [42]. U.S. EPA is expecting to issue a final regulatory determination for perchlorate based on further advice from the National Academy of Sciences. Various states in the United States have implemented guidelines or goals ranging from 1 μg/L to 18 μg/L for perchlorate in drinking water (Table 4).

In 2009, a study was conducted to estimate the national cost implications of setting a federal maximum contaminant limit (MCL) for perchlorate at levels between 4 and 24 μg/L [43]. The results showed that only 3.4% of public water systems (PWS) would be affected by a perchlorate MCL of 4 μg/L and less than 1% of PWSs would be required to treat their water at an MCL of 24 μg/L. At an MCL of 4 μg/L, total net present value compliance costs are estimated to be $2.1 billion. At MCL of 24 μg/L, the estimated compliance cost drops to $0.1 billion because of a small number of PWSs contaminated with perchlorate at that level.

## 7. Toxicity and Risk

### 7.1. Thyroid Function

The primary function of thyroid is production of the thyroid hormones, triiodothyronine (T_3_) and thyroxine (T_4_). Iodide is transported into thyroid follicular cells against a concentration gradient by the sodium iodide symporter (NIS) molecule. Once gaining entry to the thyroid follicular cells, iodide is subsequently oxidized to iodine by enzyme thyroglobulin peroxidase (TPO). Iodine is coupled to tyrosine residues on the thyroglobulin (T_g_) molecule [45]. Thyroglobulin is stored within a cavity inside the thyroid follicle in the form of a viscous substance called colloid. In response to signals from the pituitary, T_g_ is transported back into follicular cell and is cleaved to yield T_3_ and T_4_. Secretion of thyroid hormones is controlled by a well-known feedback mechanism. When serum T_3_ and T_4_ levels are too low, thyrotropin releasing hormone (TRH) is secreted by the hypothalamus and thyroid stimulating hormone or thyrotropin (TSH) is released by anterior pituitary to promote thyroidal iodide uptake and thyroid hormone synthesis. The subsequent rise in serum T_3_ and T_4_ levels result in a negative feedback, causing a reduction in circulating levels of TRH and TSH. Measuring serum levels of these hormones represents the standard approach for assessing thyroid function [46].

### 7.2. Perchlorate Toxicity

Perchlorate in drinking water or ingested by other means leads to inhibition of uptake of iodine (as iodide) into thyroid. The perchlorate ion blocks the NIS protein that normally acts as an iodide pump on the surface of the thyroid follicle. This is a competitive inhibition and therefore reversible when exposure to perchlorate ceases. Hence the adverse effect is hypothyroidism. Perchlorate also affects other tissues where NIS is known to be present in the lactating breast epithelium, gastrointestinal tract, placenta, skin and mammary gland. The presence of perchlorate decreases the synthesis of circulating thyroid hormones in the adult and decreases their placental transfer to the fetus in pregnant women. Iodide uptake inhibition is considered the mode of action for perchlorate. The mode of action agreed to by toxicologist and risk assessors identifies that inhibition of iodine uptake by perchlorate in thyroid will decrease serum T_3_ and T_4_ and increase TSH levels resulting in thyroid hypertrophy or hyperplasia, which ultimately would lead to hypothyroidism. The mode of action also identifies that perchlorate has a threshold for effects and that the degree of effects are dependant on dosage. The effects of perchlorate on thyroid function require large doses. Animal and clinical studies conducted, were mostly based on this mode of action for perchlorate.

### 7.3. Animal Toxicity Studies

To determine the health effects of perchlorate in humans, fairly extensive animal toxicity studies have been conducted. The amounts of perchlorate used in these animal studies are huge, relative to potential human exposure. In a number of sub-chronic perchlorate studies, increased thyroid follicular cell hypertrophy and hyperplasia were observed in some of the treated animals [46–48]. These data indicate that oral administration of perchlorate induces hyperplasia in the thyroid of rodents and if the exposures are lengthened, some of the lesions might progress to cancerous thyroid tumours [49,50]. Thyroid follicular tumours were observed in the F_1_ generation during a two-generation study in Sprague–Dawley rats [47]. While these studies suggest the potential for thyroid carcinogenicity associated with disruption of thyroid physiology, evidence of an early onset of tumourigenesis in the offspring of treated dams (female ancestors) requires attention because of the suggestion for potential adverse effects in the fetus. The genotoxicity studies have established that ammonium perchlorate does not have significant mutagenic activity [51,52].

There are no chronic exposure studies available in animals. There is clear evidence that perchlorate exposure at elevated doses does produce tumours, and it should be noted that perchlorate is carcinogenic in laboratory animals. The mode of action of perchlorate is established to be operative in laboratory animals and is considered relevant to humans. Rats and human thyroid work similarly and rat toxicity studies are valuable tools for qualitative information regarding the human thyroid. However, there are physiological differences between the human and rat pituitary thyroid axis, which make rats inappropriate for quantifying predicted changes in humans for risk assessment purposes. In humans, the pool of thyroxine-binding-globular thyroid hormone acts as a stable reserve to be called upon when required to supplement free thyroxine. Rats, which have no high affinity carrier do not have this reserve capacity, and compensate for this by increased TSH production and a higher rate of turnover of thyroid hormones [47].

### 7.4. Human Toxicity Studies

Humans who receive administered doses of perchlorate demonstrate rapid absorption that results in the appearance of peak serum concentrations within approximately three hours. The half-life of perchlorate in human serum is about 6 to 8 hours. Perchlorate is rapidly eliminated unchanged from the body via urinary excretion. Various studies have described the effects of perchlorate on thyroid function in healthy adult humans. In a study by Brabant *et al*. [53] five healthy men were each given daily doses of 13 mg/kg of potassium perchlorate. No differences were noted for T_4_ or T_3_ at the termination of each four-week exposure period. Brabant *et al*. [53] concluded that there was a change in iodine uptake during the perchlorate treatment. In a second study, Lawrence *et al*. [54] recruited 9 healthy men and had them ingest 10 mg/L of potassium perchlorate daily for fourteen days. There were no notable changes in T_4_, T_3_ or serum free TSH during the period of perchlorate exposure. Lawrence *et al*. [55] expanded their earlier perchlorate exposure study by changing the ingestion exposure for eight men to 3 mg of potassium perchlorate daily, again for fourteen days. During perchlorate treatment, radioiodide uptake was found to decrease in its value. Again there were no notable changes in T_4_, T_3_ or serum free TSH. Braverman *et al.* [56] observed that a six month exposure to perchlorate by thirteen healthy volunteers at doses up to 3 mg/d had no effect on thyroid function. The most comprehensive study of the kinetics of uptake of iodide and inhibition by perchlorate in humans has been described by Greer *et al*. [41]. In this study, 21 healthy women and 16 healthy men were given potassium perchlorate at several doses ranging from 0.007 mg/kg-day to 0.5 mg/kg-day for fourteen days. Greer *et al*. [41] identified the lowest exposure level (0.007 mg/kg-day) as a no observed adverse effect level (NOAEL) for exposure to perchlorate. There was no evidence for changes in free serum T_4_, T_3_ or TSH in any of the participants at doses less than 0.5 mg/kg-day during the study. Blount *et al.* [57] assessed the effect of low-level exposure to perchlorate on thyroid function in 2,299 men and women in the United States during 2001–2002. The study evaluated the potential relationship between urinary levels of perchlorate and serum levels of TSH and T_4._ The results indicated a small increase in serum TSH and small decrease in serum T_4_ only in women with urine concentrations < 100 μg/L. Perchlorate was found to have no effect on TSH and T_4_ in men. In contrast, Pearce *et al.* [58] found no change in thyroid function in first trimester pregnant women in Europe whose iodine intake was < 100 μg/L. Another study used individuals who received occupational exposures to perchlorate [59]. This study compared workers and members from the surrounding community (non-exposed) at an ammonium perchlorate manufacturing facility. A total of twenty-nine perchlorate workers and twelve community controls participated in the study. Exposure doses were predicted for each shift from assumed 8-hour half-life of perchlorate. It was reported that worker radioiodide uptake values measured during exposure periods were significantly lower than their baseline pre-exposure values. Thyroid hormone levels showed no differences in TSH but higher concentrations of T_3_ or T_4_ for post-shift exposure was observed.

### 7.5. Ecotoxicity Studies

In contrast to the abundance of human health data, few ecological data exist on the potential exposure and effects of perchlorate in the environment [60]. There are especially few data regarding the presence and effects of perchlorate on invertebrates, such as earthworms. The toxicity of sodium perchlorate to *Eisenia fetida* was evaluated in sub-chronic tests in artificial soil. The sub-chronic (21 day sand tests) EC50 for sodium perchlorate to *E. fetida* reproduction (cocoon production) was 1.3 μg/g. The sub-chronic (28 day artificial soil tests) EC50 for sodium perchlorate to *E. fetida* reproduction (cocoon production) was 350 μg/g [61]. To investigate chronic toxicity in fishes, mosquitofish (*Gambusia holbrooki*) adults and fry were exposed to aqueous sodium perchlorate at 1, 10, and 100 mg/L, and growth and reproductive performance were determined [62]. Five-day acute toxicity tests were also performed. The LC_50_ of sodium perchlorate was 404 mg/L. Growth was enhanced at 1 mg/L but inhibited at 10 mg/L. These results suggest that, at environmentally relevant concentrations, perchlorate does not induce acutely toxic effects but may have mild stimulatory or hormetic effects on fitness parameters in this species. Adult female and male zebrafish were paired in water containing ammonium perchlorate at 0, 18, and 677 mg/L for up to 8 weeks [63]. Perchlorate does not accumulate in whole fish. Thyroid follicle hypertrophy and angiogenesis were observed for the 677 mg/L treatment group for 4 weeks, which may be due to extra thyroidal toxicity. More pronounced effects including hypertrophy, angiogenesis, hyperplasia, and colloid depletion were observed for the 18 mg/L treatment group for 8 weeks. Goleman *et al*. [64] evaluated the effects of ammonium perchlorate at environmentally relevant concentrations on development and metamorphosis in amphibian, *Xenopus Laevis* after 70-day exposure. No concentration-dependent developmental abnormalities were observed below the 70-day LC_50_ (223 mg/L). Delays in metamorphosis in *Xenopus laevis* at concentrations ranging from 0.005 to 0.1 mg/L and inhibition of metamorphosis at 0.147 mg/L have been reported. It was concluded that perchlorate may pose a threat to normal development and growth in natural amphibian populations. Birds whose migratory routes transect contaminated watersheds or those whose movement is restricted may be exposed to perchlorate. Thyroid glandular T4 content was significantly decreased in 21-day bobwhite quail embryos from eggs with a concentration of ≥ 0.5 μg/g ammonium perchlorate and in hatchlings from eggs with ≥ 50 mg/L ammonium perchlorate [65]. However, egg concentrations of up to 300 μg/g did not result in hypothyroidism in embryos or hatchlings. Perchlorate sensitivity also appears to be species specific in birds [66]. In areas where perchlorate-contaminated groundwater is used for irrigation and/or a source of drinking water, livestock may consume considerable amounts of perchlorate on a daily basis. Perchlorate exposure in cattle was evaluated by monitoring heifer calves on a site with access to perchlorate contaminated water (25 μg/L) for 14 weeks. Perchlorate was detected in blood twice (15 μg/L and 22 μg/L) in one of the heifer calves at week 4 and week 6, respectively [67].

## 8. Natural Biodegradation

Aslander [68] was the first to observe microbial degradation of chlorate in soils. Later, it was shown that bacteria capable of perchlorate and chlorate respiration exist in nature [69,70]. van Ginkel *et al.* [70] demonstrated qualitatively that chlorate respiring microorganisms were present in a variety of environments such as wastewaters, rivers, sediments and soils. These studies suggest that chlorate and perchlorate are biodegradable under certain conditions. Several bacterial isolates have been shown to be capable of completely reducing perchlorate to chloride for cell respiration. The details of perchlorate reducing strains and the reduction process are discussed in detail under the treatment technologies section. Although, perchlorate respiring bacteria are widely distributed in nature, perchlorates still persist in the environment. Intrinsic remediation of perchlorate contaminated site is possible if perchlorate respiring microorganisms exist naturally in the system and can compete for existing sources of organic matter. Otherwise, perchlorate reduction must be enhanced by addition of nutrients to the contaminated sites. Wu *et al.* [71] observed that the presence of perchlorate in the soil tested appears to have enriched the number of perchlorate and chlorate respiring microorganisms in that soil. Perchlorate respiring microorganisms were found to be present in sufficient numbers to achieve complete perchlorate degradation using lactate in soil microcosms in only 7 days.

## 9. Treatment Technologies

Widely used treatment technologies used for removing perchlorate from groundwater include, ion exchange, use of bioreactors and in-situ bioremediation. Groundwater contamination of perchlorate is of concern than contamination in soil due to the highly soluble nature of perchlorate in water. Hence the following discussions apply to groundwater and drinking water treatment.

### 9.1. Ion Exchange (IX)

Ion exchange (IX) is an *ex situ* technology used to remove perchlorate from groundwater and surface water. The most commonly used ion exchange media are synthetic, strongly basic, anion exchange resins. Extensive research has focused on developing IX resins that would selectively remove perchlorate when the water contains other competing anions, such as chloride, sulphate, nitrate and bicarbonate, at higher concentrations. Strong base anion-exchange resins have proven to be very effective in removing perchlorate from water [72]. The functional groups in perchlorate resins have positive charges, which are initially dosed with anions such as chloride ion that attaches itself to these positively charged functional groups. The perchlorate anion in the influent water is also attracted to the positively charged functional groups, due to its stronger attractive force and replaces the chloride ion [73]. Thus ion exchange removes the perchlorate ion from the aqueous phase by exchange with the chloride ion, populating the treated water with chloride ions. The regeneration solutions used to regenerate the exhausted resin include 12% brine solution of sodium chloride, which can recapture approximately 7% of the perchlorate from the resin, and ferric chloride in solution with hydrochloric acid, which can recapture close to 100% of the perchlorate from the resin [74]. Many groundwater and drinking water systems incorporate ion-exchange to treat water containing 10 to 10,000 μg/L perchlorate (Table 5). There are fifteen full scale applications of ion exchange so far to remove perchlorate.

### 9.2. Bioreactors

A bioreactor is an *ex situ* biological treatment system for degrading perchlorate from contaminated groundwater and surface water using microorganisms. Certain bacteria can reduce perchlorate in soil and water under certain anaerobic conditions. The microorganisms most often used in perchlorate treatment are facultative anaerobes that use electron acceptors such as nitrate, sulphate and perchlorate to reduce organic compounds. In addition to the electron sink, these organisms require carbon sources such as ethanol, methanol, or acetic acid for growth. Groundwater naturally contains some perchlorate reducing bacteria [8] and under reducing conditions such as in a bioreactor, population of such bacterial species will increase over time as the microbes select perchlorate as the dominant electron acceptor. Addition of carbon sources induces the microorganisms in water to extract electrons from the carbon source and deliver them to perchlorate. This electron transfer induces biotransformation of perchlorate ion to chlorate and then chloride [2]. Recent research shows that perchlorate reduction involves a three step process catalyzed by two enzymes. A perchlorate reductase enzyme catalyzes reduction of perchlorate to chlorate and then to chlorite. A chlorite dismutase enzyme causes further breakdown of chlorite to chloride and oxygen [77,78]. The rate limiting step is believed to be perchlorate reduction to chlorate [79]. Perchlorate reducing strains reported in the literature include *Vibrio dechloraticans* Cuznesnove B-1168 [80], *Wolinella succinogens* HAP-1 [81], and isolates GR-1 [82], perclace [61], *Dichlorosoma* sp. KJ [83] and *Dechloromonas agitata* strain CKB [84]. Several different types of bioreactors have been proposed to remove perchlorate from water using mixed and pure cultures of bacteria [85]. Table 6 gives a summary of various bioreactors used for treating perchlorate containing water. Fluidized bed reactors (FBR) and packed bed reactors (PBR) are two types of bioreactors widely used for removal of perchlorate. FBRs are generally made up of suspended sand or granular activated carbon media to support microbial activity and biomass growth. The biomass on the packing is kept uniform in the reactor by mixing, and biomass thickness is controlled by shear and particle collisions. Packed bed reactors are made up of static sand or plastic media to support the growth of microbes. Perchlorate has been degraded in saturated-flow packed bed reactors, air lift reactors and unsaturated flow packed bed reactor [87,88]. The overall rate of perchlorate degradation in these reactors is first order with respect to log mean perchlorate concentration in the reactor [85]. The highest perchlorate removal rate in a fixed bed reactor was obtained for a sand packed reactor using *Dechlorosoma* sp. KJ [83]. Studies have been conducted on the use of hydrogen gas reactors, which use continuous hydrogen gas transfer to the biofilm with unsaturated flow conditions for autotrophic perchlorate reduction [88,89]. Hydrogen oxidizing bacterial isolate, designated as JM has been used in these studies.

### 9.3. In situ Biodegradation

*In situ* biodegradation (ISB) can be used for source reduction, dissolved phase contaminant reduction or as a biological barrier to contain a plume [93]. ISB involves delivery of nutrients such as ethanol, lactate, acetate, citrate, sugars and edible oils to the subsurface to promote the biodegradation of perchlorate by the indigenous bacteria. The most important consideration in ISB is the ability to transmit and mix liquids in the subsurface [93]. Major system components include extraction wells, conveyance piping, electron donor dosing station, electron donor delivery/recharge wells, barrier wall width and depth, barrier components and performance monitoring wells. Electron donors can be delivered to the aquifer through a variety of active (recirculation) or passive (injection or trenching/barrier) methods [2]. Permeable reactive barriers (PRB) can be used to deliver the amendments to the contaminated groundwater. PRBs are installed as permanent, semi-permanent, or temporary units across the flow path of the contaminant plume. Contaminants in the groundwater that flow through a PRB are degraded chemically or biologically. This biobarrier approach can use engineered trenches or barriers containing solid phase [94] or viscous substrates [95] placed across the flow path through direct push injections. Examples of reactive materials used include edible oils, pecan shells, cotton seed, chitin, limestone, and other composting materials. Another approach to deliver electron donors include mobile amendment systems, which are characterized by injecting less viscous water soluble substrates into the up-flow of the contaminant plume, allowing the substrate to move down treating the groundwater as it moves. Active treatment can use groundwater extraction and reinjection system to distribute substrate in the subsurface [96] or horizontal-flow treatment wells to mix and distribute electron donors in groundwater [76]. Table 7 provides a list of ISB technology used for treating perchlorate contaminated water.

### 9.4. Other Processes

1. Adsorption: Adsorption involves the use of adsorbent media such as granular activated carbon (GAC) or activated alumina to remove perchlorate. For sites that have groundwater containing contaminants other than perchlorate, previous experience shows the potential for the use of treatment trains consisting of standard GAC/ion exchange resins or tailored GAC [97]. Cationic surfactant-tailored activated carbon has been studied to remove perchlorate in a laboratory scale column test; perchlorate was removed to below detection levels and increased the breakthrough time up to 30 times longer than standard activated carbon [98].
2. Membrane filtration: Membrane filtration treatment includes reverse osmosis (RO), nanofiltration (NF), ultrafiltration (UF) and electrodialysis (ED). Three main types of semi-permeable membranes have been evaluated for the treatment of perchlorate in groundwater which includes high pressure RO membrane, nanofiltration membrane and low pressure RO membrane. High pressure RO membranes (> 150 psi) have been observed to remove about 99.9% of perchlorate [99]. NF membranes have a nominal pore size of approximately 0.001 micron and require an operating pressure in the range of 80 to 150 psi. NF membranes have been found to allow particles such as salts to pass through them. UF membranes have a pore size of approximately 0.002 to 0.1 microns and operate at a lower pressure (30 to 100 psi). Tight UF membranes, having smaller pores than UF but larger than NF, have been found to be ineffective in treating perchlorate [2]. Liang *et al.* [100] conducted pilot scale studies to evaluate the performance of perchlorate removal by production sized RO, NF and UF membranes. RO and NF membranes were observed to consistently remove more than 86.7% of perchlorate while tight UF membranes proved to be ineffective. Addition of cationic surfactant to UF membrane did not prove to be effective; this resulted in plugging the membrane.
3. Electrochemical reduction: Electrochemical reduction of perchlorate anion has been studied in a cell with a nickel working electrode and a platinum counter electrode in concentrated solutions of hypochlorous acid [102]. Lee and Kramer [103] examined titanium metal as a chemical reductant to remove perchlorate in water. The activation of titanium was achieved by eliminating the localized surface oxide film using electrochemically induced pitting corrosion. It was observed that a higher current increases the activity of pitting corrosion. This dissolves a higher concentration of transitory titanium metal ions in the vicinity of the pits, which results in a higher rate of perchlorate reduction. The surface of the bare Ti(0) inside the pits induces further electrochemical reactions and causes faster rate of chloride oxidation to chlorine by increasing the current.
4. Phytoremediation: Phytoremediation is an in situ mechanism by which vegetation is used to treat perchlorate. The most successful plants in perchlorate degradation include French terragon, cottonwood, and willow. Under hydroponic conditions willows were observed to degrade perchlorate from 10,000 μg/L to below detection in about 53 days [9]. Other plants that have been successfully tested include salt cedar trees, bulbrushes, cattails and sedges. Perchlorate concentrations have decreased from 20 μg/L to less than 4 μg/L using constructed wetlands [2].
5. Use of iron particles: Stabilized elemental iron nanoparticles have been studied to remove perchlorate [104]. The results showed that temperature played a critical role in perchlorate degradation process. Approximately 100% of perchlorate was transformed to chloride during the reaction without any detectable intermediate products. Kinetics of perchlorate reduction by elemental iron was examined at elevated temperatures using microwave heating and conventional block heating [105]. Results from microwave heating study showed that 98% of perchlorate was removed in one hour at 200 deg C. Similar results observed with block heater indicated that enhancement in the rate of perchlorate removal by elemental iron was mostly due to heat energy at a higher temperature. Gurol and Kim [106] investigated chemical reduction of perchlorate using iron and iron oxide under various conditions. The main result of their investigation was that the addition of metallic iron (100 g/L) combined with exposing the solutions to ultraviolet light at wavelengths < 185 nm achieved 77% reduction in perchlorate.
6. Catalytic reactors: A preliminary study using a catalyst that efficiently uses hydrogen gas to reduce perchlorate completely to chloride has been reported [107]. The catalyst has been prepared by the addition of methylthrioxorhenium to a combination of 5% Pd-carbon powder. Initial batch studies have indicated complete reduction of perchlorate to chloride.

Studies such as thermal destruction, capacitative deionization have also been reported in literature as effective treatment systems for perchlorate.

## 10. Conclusions

Perchlorate has been found to be widespread in drinking water sources in the United States and a few other countries. Recent studies have confirmed the presence of perchlorate in Canadian surface waters. Though, known adverse effect of perchlorate exposure on humans is hypothyrodism, detailed animal toxicity studies conducted on two generation of rats have shown to have potential adverse effects on their fetuses. Perchlorate has been found in cow’s milk and mother’s milk and hence perchlorate ingestion by infants through maternal intake is high. Therefore, perchlorate can be considered as a contaminant of concern in the near future in Canada.

The most common perchlorate treatments currently in use employ ex situ technologies. Two treatment technologies currently dominate the field of perchlorate treatment: ion exchange and biological treatment. Research demonstrated that bioreactors, mostly fluidized bed reactors operated under anaerobic conditions, could effectively reduce high concentrations of perchlorate. Selective ion exchange resins of various types have been developed to treat lower concentrations of perchlorate. Although bioreactors have been proven to be effective, the best applicable technology for removal of perchlorate remains to be ion exchange. Ion exchange resins are used for treatment of drinking water supplies and groundwater containing 10 to 100,000 μg/L perchlorate. Research is presently directed on developing technologies that could remove perchlorate to less than 10 μg/L from water sources. Such recent technologies include electrochemical reduction, catalytic reduction and use of metallic nanoparticles.

## Figures and Tables

**Table 1.** Characteristics and uses of some perchlorate compounds [2,3].

**Compound**	**Formula**	**Molecular weight (g/mol)**	**Density (g/cm^3^)**	**Physical appearance**	**Aqueous solubility at 20° C (10^3^mg/L)**	**Decomposition temperature (°C) / Reaction**	**Uses**
Ammonium perchlorate	NH_4_ClO_4_	117.488	1.952	Colourless or white orthorombhic and regular crystals	217–220	150	Energetic booster in rocket fuel.
Sodium perchlorate	NaClO_4_	122.439	2.02–2.499	Hygroscopic/deliquescent, white orthorombhic crystals	2010	492	Strong oxidizing agent used in explosive and chemical industries
Potassium perchlorate	KClO_4_	138.547	2.5298	Colourless crystal to white crystalline powder, hygroscopic	7.5 to 16.8	440	Solid oxidant for rocket production.
Litium perchlorate	LiclO_4_	106.3906	2.428–2.429	Deliquescent, white crystal	375	< 250–400	Electrolyte in voltaic cells, synthesis of organic chemicals
Perchloric acid	HClO_4_.2H_2_O	223.21	2.21	White, hygroscopic powder	Very soluble	250	Analytical, oxidizing and dehydrating agent.

**Table 2.** Releases of perchlorate compounds [3,26,27].

Application	Potential release	Remarks
Agricultural uses	Chilean nitrates and potash ores	App. 75,000 tons fertilizer containing 0.01 wt% perchlorate used annually between 2002 and 2004.
Solid propellants	Disposal of solid propellants, untreated liquid waste from hog out process, space shuttle and other solid fuel launch vehicles.	Perchlorate containing debris, scraps of solid propellants, rejected rocket motors not burned to completion.
Munitions	Hydraulic washout of equipment in munition manufacturing, corrosion and subsequent degradation of casings. Expended munitions and simulators.	Systems containing perchlorate include fuses, flares, illumination rounds, simulators, grenades, etc. Improper functioning/ incomplete propellant consumption and subsequent leaching.
Fireworks	Firework based perchlorate residue	Contain up to 70 wt% potassium or ammonium perchlorate.
Safety and hazard flares	Precipitation from flame manufacturing sites	Preliminary research suggests unburned and burned flares lead to 3.6 g and 1.9 g, respectively of perchlorate. Residue from burned flare containing leachable amount of 2000 μg/flare perchlorate [25].
Matches Commercial explosives	Release likely to be at production facility Use of unlined ponds to collect production derived wastewater in explosive manufacturing and accumulation of perchlorate containing sludge. Disposal of perchlorate containing wastes at open burn/open detonation resulting in perchlorate residue generation. Black powder manufacturing, storage leakage and disposal of bags and containers of perchlorate in landfill. At blasting site if detonation is incomplete.	- Contain up to 30 wt% sodium/ammonium/potassium perchlorate as chemical sensitizing agent.
Industrial uses	Release likely from production facility and handling of perchlorate containing compounds.	Uses include: Bleach activators in detergents, constituent in rust removers, perchloric acid for airbag inflators and processing rare earth element ores.
Laboratory	Detergent based lab glassware cleaning agents such as Alconox, Liquinox have found to have up to 2.5 mg/kg perchlorate [2].	Use in labs in industries, academia and defense settings. Groundwater contamination found to be associated with actinide research, high explosive synthesis and testing [26].
Sodium chlorate production	-	Electrochemical production of sodium chlorate can generate perchlorate as an impurity.

**Table 3.** Food supplies found to be contaminated with perchlorate.

Food studied	Reference
Drinking water (USA, Japan, China)	[3,23,24]
Lettuce (concentrations ranging from 0.5 to 129 μg/kg in 116 of 127 samples)	[27]
Laboratory studies on lettuce, tobacco, soybeans, alfalfa, tomato, cucumber	[28–30]
Field studies on lettuce, winter wheat crops, alfalafa, watercress, chinaberry and mulberry trees, cucumber, cantaloupe and tomatoes.	[29–31]
Cow’s milk (average perchlorate concentration 5.76 μg/L)	[27]
Cow’s milk (concentration ranging from 0.47 to 11 μg/L)	[32]
Human milk	
(concentration ranging from 1.4 to 92.2 μg/L in 18 states, USA)	[29]
(concentration ranging from 1.3 to 411 μg/L in Boston area, USA)	[34]

**Table 4.** Summary of proposed state drinking water standards or action levels for perchlorate as of March 2006.

Country/State	PHG/MCLG (μg/L)	Proposed standard (μg/L)	Action level (μg/L)	Remarks	Reference
Canada			6	Guidance value	[39]
USEPA			15	Interim drinking water health advisory level	[43]
Arizona			14	Based on child exposures	[44]
Oregon			4	-	[44]
California	6		6	Notification level	[44]
New Jersey	5			Health based value	[2]
Maryland			1	Advisory level	[44]
Massachusetts		2	1	Advisory Level for children and other at-risk populations for Bourne	[44]
Nevada			18	Public notice standard	[44]
New Mexico			1	Drinking water screening level	[44]
New York			5	Drinking water planning level	[44]
			18	Public notification level	
Texas			17 51	Residential protective clean up level (PCL) Industrial/commercial PCL	[44]

PHG - Public health goal

MCLG - Maximum contamination level goal

**Table 5.** A summary of performance of ion exchange used for treatment of perchlorate.

Source of contamination	Description	Performance	Reference
Ground water (Presence of nitrates and chlorinated solvents).	A non-regenerable Perchlorate-selective resin is used. The system operating flow rate 400 gpm.	Initial concentration 50 μg/L. Effluent concentration < 4 μg/L	[75]
Drinking Water	A fixed bed, non-regenerable anion-exchange resin is used.	NA	[76]
Groundwater (Plant Ion Exchange System (PIES) and Wash Ion Exchange System (WIES))	The PIES included twelve single-use anion exchange columns in 4 parallel trains. The WIES included three single-use ion exchange columns in series.	Initial concentration 80,000 to 350,000 μg/L. Effluent concentration 500 to 2,000 μg/L.	[75]
Drinking Water	A fixed bed, non-regenerable anion exchange system is used. The system consists of 10 ion exchange vessels, each loaded with a strong-base, quarternary amine resin, operating at 6,000 gpm.	Initial concentration 20 to 50 μg/L. Effluent concentration < 4 μg/L.	[75]
Groundwater	A non-regenerable, nitrate- selective anion exchange system is used. Operating flow rate 10,000 gpm.	Initial concentration 15 μg/L. Effluent concentrations < 4 μg/L.	[75]
Groundwater (other contaminant trichloroethane)	Six fixed bed, non-regenerable anion exchange systems are used.	Initial concentration 20 μg/L. Effluent concentration < μg/L	[75]

**Table 6.** Summary of Bioreactor studies for perchlorate treatment.

Type of system	Description	Concentration (μg/L)	Reference
Upflow fluidized bed reactor	Water contaminated with perchlorate, nitrate and chlorinated solvents. The system consists of bioreactor, an UV oxidation system, an air stripper and a disinfection system. The bioreactor uses ethanol as feed.	Influent: 2,500 Effluent from bioreactor: < 4	[75]
Fluidized bed reactor	System consists of four primary and four secondary FBRs, using sand and GAC as media.	Influent: 200,000 Effluent: < 18	[76]
Fluidized bed reactor	Water contaminated with perchlorate, metals and volatile organics. FBR is inoculated with pre-conditioned GAC containing biosolids acclimated to perchlorate removal. Acetic acid and inorganic nutrients are added. Excess biomass is removed from media.	Effluent: <4	[77]
Hall bioreactor	System consists of baker tank, deaeration reactor, methanol tank, and patented Hall reactor. Hall reactor contains floating media (poly- urethane based sponge) cut into one-centimeter cubes. Media provides support to bacterial colonies. Methanol fed as carbon source. Temperature ranges from 8 to 35 deg C.	Influent: 300 to 1000 Effluent: non-detectable limits	[86]
Upflow packed bed reactor	Use of a packed bed anaerobic bioreactor containing sand or plastic media. Acetic acid was constantly fed. Biological reactions initiated by bioaugmentation of the columns with perchlorate respiring bacterial strain *Dechlorosoma* sp.	Influent: 75 Effluent: < 4	[90]
Hollow fiber membrane bioreactor	The bioreactor consists of a bundle of hydrophobic hollow fiber membranes (polyurethane encased within microporous polyethylene) where hydrogen gas diffuses through and autotrophic biofilm is developed outside the membrane, where bacteria (*dichloromonas* sp. PCI) reduce perchlorate.	Influent: 55 Effluent: 2	[91]
Packed bed bioreactor	Laboratory scale treatment of groundwater contaminated with perchlorate and nitrate. The reactor was fed with a gas mixture of H_2_ and CO_2_ and inoculated with hydrogen oxidizing perchlorate degrading bacterium.	Influent: 75 Effluent: 25–30% reduction.	[89]
Packed bed bioreactor	A pure perchlorate respiring isolate (KJ) and mixed culture was used. Acetate required by mixed culture was observed to be twice more than that required by pure culture. Detention time observed to reduce using pure isolate.	Influent: 20 Effluent: < 4	[83]
Anaerobic treatment	Domesticated sludge was used. Acetate was fed as carbon source. Bacteria exposed to DO were incapable of reducing perchlorate. Addition of little Fe(0) accelerated while Fe(II) inhibited perchlorate removal.	Reduced to non-detectable limits.	[92]

**Table 7.** Summary of in situ bioremediation for treating perchlorate.

Type of system	Material injected	Description	Perchlorate concentration	Reference
Injection	Corn syrup, ethanol	Substrates were used to flood the vadose zone and drive it anaerobic.	Reduction from 5000 to 500 μg/kg	[75]
Injection	Emulsified Soy bean oil	A portion of the oil is trapped within the soil pores leaving a residual oil phase to support anaerobic biodegradation.	Initial: 10,000 μg/L Final: 4 μg/L	[101]
Barrier	Mushroom compost, soybean oil, wood chips	A series of trenches containing the substrates mixture. Shallow trenches are cut into limestone and designed to capture ground water and run off.	Initial 13,000 μg/L Final: below detection limits	[76]
Passive Injection	Lactate	Buffer added to the aquifer to increase ground water pH.	Over 95% reductions.	[76]
Ground water recirculation	Citric acid	Recirculation design consists of single groundwater extraction well and rejection well. Chlorine dioxide used to reduce biofouling.	Initial: 530000 μg/L Final: < 4 μg/L	[75]
